# *Go*!: results from a quasi-experimental obesity prevention trial with hospital employees

**DOI:** 10.1186/s12889-016-2828-0

**Published:** 2016-02-19

**Authors:** Lara J. LaCaille, Jennifer Feenstra Schultz, Ryan Goei, Rick A. LaCaille, Kim Nichols Dauner, Rebecca de Souza, Amy Versnik Nowak, Ronald Regal

**Affiliations:** Department of Psychology, 1207 Ordean Ct., University of Minnesota Duluth, Duluth, MN 55812 USA; Department of Economics, 1318 Kirby Dr., University of Minnesota Duluth, Duluth, MN 55812 USA; Department of Communication, 1121 University Dr., University of Minnesota Duluth, Duluth, MN 55812 USA; Department of Health, Physical Education and Recreation, 1216 Ordean Ct., University of Minnesota Duluth, Duluth, MN 55812 USA; Department of Mathematics, 1117 University Dr., University of Minnesota Duluth, Duluth, MN 55812 USA

**Keywords:** Obesity, Prevention, Worksite, Cafeteria, Diet, Physical activity

## Abstract

**Background:**

Worksite obesity prevention interventions using an ecological approach may hold promise for reducing typical weight gain. The purpose of this study was to examine the effectiveness of *Go!*, an innovative 12-month multi-component worksite obesity prevention intervention.

**Methods:**

A quasi-experimental non-equivalent control group design was utilized; 407 eligible hospital employees (intervention arm) and 93 eligible clinic employees (comparison arm) participated. The intervention involved pedometer distribution, labeling of all foods in the worksite cafeteria and vending machines (with calories, step equivalent, and a traffic light based on energy density signaling recommended portion), persuasive messaging throughout the hospital, and the integration of influential employees to reinforce healthy social norms. Changes in weight, BMI, waist circumference, physical activity, and dietary behavior after 6 months and 1 year were primary outcomes. Secondary outcomes included knowledge, perceptions of employer commitment to employee health, availability of information about diet, exercise, and weight loss, perceptions of coworker support and frequency of health discussions with coworkers. A process evaluation was conducted as part of the study.

**Results:**

Repeated measures ANCOVA indicated that neither group showed significant increases in weight, BMI, or waist circumference over 12 months. The intervention group showed a modest increase in physical activity in the form of walking, but decreases in fruit and vegetable servings and fiber intake. They also reported significant increases in knowledge, information, perceptions of employer commitment, and health discussions with peers. Employees expressed positive attitudes towards all components of the Go! intervention.

**Conclusions:**

This low-intensity intervention was well-received by employees but had little effect on their weight over the course of 12 months. Such results are consistent with other worksite obesity prevention studies using ecological approaches. Implementing low-impact physical activity (e.g., walking, stair use) may be more readily incorporated into the worksite setting than more challenging behaviors of altering dietary habits and increasing more vigorous forms of physical activity.

**Trial Registration:**

This study was registered with clinicaltrials.gov (NCT01585480) on April 24, 2012.

**Electronic supplementary material:**

The online version of this article (doi:10.1186/s12889-016-2828-0) contains supplementary material, which is available to authorized users.

## Background

Increasing obesity rates over the past three decades have resulted in considerable efforts to find effective interventions to curb this trend. Until recently, strategies to combat obesity have focused largely on identifying and treating overweight and obese persons through individual and/or group behavior weight loss programs. Although such interventions may have an effect on participants, the unfortunate pattern is that only a small percentage of overweight/obese people take part in these programs and weight losses are typically not sustained [1, 2]. Consequently, there has been a call to develop more social ecological approaches that address social and environmental factors (i.e., the “obesogenic environment”) to preventing weight gain [3]. Interventions that use an ecological framework not only convey information and education, but seek to change the environment so that healthy eating and physical activity become the default choices. Moreover, weight gain prevention programs that emphasize a broad reach, targeting large segments of the population rather than select individuals, are critical if we hope to have a meaningful impact on the obesity epidemic.

Worksites offer an opportune setting in which to target obesity prevention efforts because most employed adults spend about half of their waking hours at work [4], and worksites have a number of characteristics that could support multi-component, ecological interventions. For example, worksites typically offer existing social networks, formal communication systems, readily available eating environments, and access to employer support programs. A meta-analysis of worksite nutrition and physical activity interventions that examined weight/body mass index (BMI) outcomes found that such programs achieved modest changes in weight (mean weight loss = 2.8 lbs) [5]; though few of these programs specifically targeted body weight as the primary outcome (and those that did tended to target only overweight/obese employees), and few interventions included social or environmental components.

In response to the paucity of clinical trials examining ecological approaches to weight gain prevention programs in the worksite, the National Heart, Lung, and Blood Institute (NHLBI) sponsored seven large scale randomized clinical trials in 2004 [6]. To date, four of these studies have published their main outcome findings [7–11]. Overall, findings from the few published studies that used ecological approaches in the worksite have been mixed and shown small to no effects for weight gain prevention [6–12]. However, the specific components, intensities, and populations of these interventions varied considerably, suggesting that there is value in examining additional strategies that may be more effective.

The present study explored novel approaches to obesity prevention within an ecological framework via a worksite weight gain prevention program (referred to as “*Go!*”) that included a number of theoretically-driven and low cost components that targeted changing eating and physical activity behavior. The innovative components included labeling (involving calories, step equivalents, and traffic lights) of all foods in the cafeteria and vending machines, and the identification and use of influential employees to target social norms. This paper describes the design, intervention components, and primary outcomes for this 12-month intervention. The Transparent Reporting of Evaluations with Nonrandomized Designs (TREND) guidelines [13] are followed in reporting on the study.

## Method

### Objectives and hypotheses

The purpose of this study was to design and evaluate a multi-component weight gain prevention program in a workplace setting. Offering individual tools, providing information and persuasive messaging, and changing the social and physical environments over 12 months were hypothesized to result in healthier eating and increased physical activity. Consequently, it was expected that employees in the intervention condition would not gain weight or would gain less weight than the comparison group.

### Study design

Using a quasi-experimental nonequivalent control group design [14], employees from a mid-sized healthcare system in the upper Midwest region of the United States were recruited. The intervention group consisted of employees from the hospital campus (including the main hospital, administrative offices, and several specialty outpatient clinics), whereas the comparison group consisted of employees from six primary care clinics located within 15 miles of the main hospital campus. This hospital system was chosen due to an existing working relationship with key hospital personnel. A non-randomized design was utilized for reasons of access, available resources, and the exploratory nature of some intervention components. Employees could not be randomized to conditions because of the nature of the intervention involving social networks and environmental changes made throughout the hospital. The primary care clinic employees were the best available comparison group as they worked autonomously from the hospital environment, yet still had the same employer (i.e., same benefits, wellness programming, health communications).

The study was approved by the University of Minnesota Institutional Review Board (reference #: 1002S78225) and the St. Luke’s Institutional Review Board (reference #: HSRC010-003). Informed consent was gathered from every participant in the study. Formative stages of the study took place in 2010, which involved identifying influentials as well as key leaders in the hospital to serve on an advisory board created to provide input on program development, implementation, and measurement. It was noted that previous wellness programs that involved sign-up and participation, such as Weight-Watchers groups, walking groups, and walking contests had poor response rates (less than 3 % of employee involvement). The intervention and associated data collection took place November 2010 to December 2011, and the data were analyzed from 2012 to 2014. This study was registered with clinicaltrials.gov (NCT01585480) on April 24, 2012.

### Participant recruitment

All hospital and clinic employees were invited to participate, and were recruited through multiple methods, including email, direct mail, newsletter, and hospital intranet link (all sent a few days before data collection began). In exchange for participating in the assessments, hospital employees received a pedometer and up to $50 cash ($10 at baseline and $20 at the 6- and 12-month assessments). Unlike studies that enroll participants into “a program,” this study primarily recruited hospital employees to “complete assessments” to evaluate the effectiveness of an obesity prevention program being implemented throughout the hospital. That is, there was no expectation that employees in the study engage in any additional activities or attempt to lose weight as part of the study, even though they were informed that the Go! program was being implemented to help employees avoid weight gain. The rationale for this was that the purpose of the study was to determine the effects of hospital-wide changes on employee eating and physical activity behavior, rather than targeting only individuals ready and wanting to lose weight. That said, at the baseline assessment, enrollees received information and a pedometer that they were encouraged, but not expected, to use. After this point, no additional intervention components were given to the enrolled participants that were not also provided to the rest of the hospital employees, other than the data collection itself. Clinic employees were similarly asked to complete assessments, though informed that it was in order to examine changes in eating, physical activity, and weight of healthcare employees over time, without mention that they were a comparison group for an intervention occurring at the main hospital. This was done in order to maintain the status quo (i.e., no new wellness opportunities) as well as to minimize perceptions of unfairness, (i.e., that clinic employees were being deprived of a special program offered only to hospital employees). They received the same financial incentive, but not the pedometer.

All employees over the age of 18 were allowed to participate, regardless of employment status or intentions to remain employed, pregnancy status/intentions, health status, or desire to lose or maintain weight. Although such an inclusive approach resulted in greater attrition, we believed that involving the largest number of employees possible would have greater impact on social norms and subsequent intervention effects. Only employees who completed the first assessment were recruited for future assessments. Participants received up to three reminders to participate in follow-up measures.

### Intervention

**Fig. 1:**
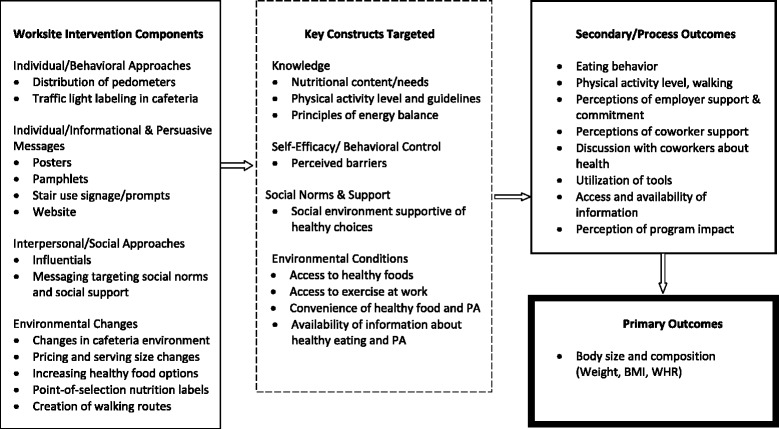
Analytic framework using the Social Ecological model for worksite interventions to prevent obesity (adapted from Anderson et al., [5])

All intervention components were delivered over 12 months and designed to increase physical activity, reduce calorie consumption, and manage weight using principles of energy balance. Specific messages and approaches were selected following a social ecological framework (i.e., targeting individual, interpersonal, social, environmental, and/or systems levels) and drew from three well-established multidisciplinary theories in health-related behavior change: extended parallel process model [15, 16], theory of planned behavior [17, 18], and social cognitive theory [19]. These theories suggest that knowledge, self-efficacy/perceived behavioral control, perceived risk, attitudes, and social norms all affect health behavior change. Each of these constructs was targeted through methods employed at the individual, interpersonal, social, and/or environmental level (i.e., use of the social ecological framework). See Fig. 1.

#### Nutrition labeling

**Fig. 2:**
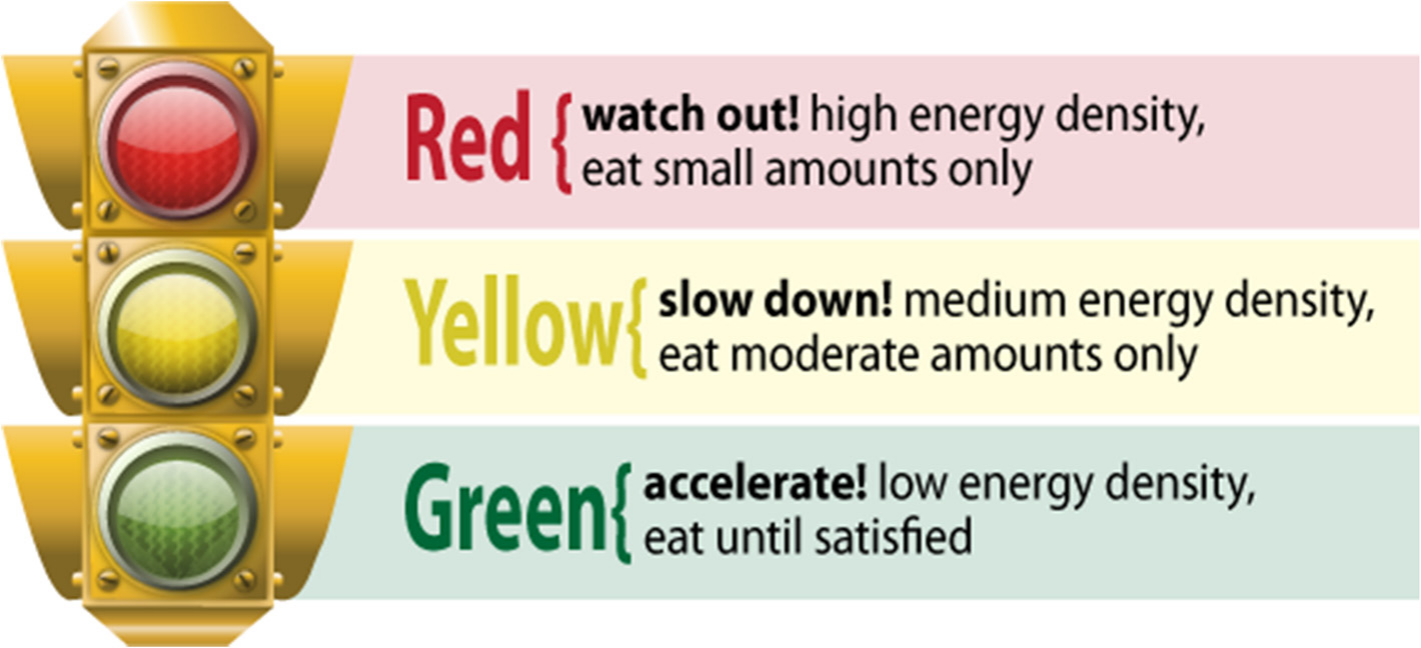
Traffic light labeling scheme

Point-of-purchase calorie labeling on menus has been recommended to increase consumer knowledge and awareness of intake [20], yet few studies have fully incorporated such interventions into cafeteria or worksite settings [21]. A number of other approaches to labeling foods have been recommended, and to some degree, tested. In Europe, the Food Standards Agency has recommended that packaged foods include color-coded “traffic lights” on the front of packages, giving color ratings (green = go, eat frequently, yellow = caution, eat in moderation, red = stop, eat only occasionally) to foods [22]. Data suggest this particular labeling scheme results in the highest rate of consumer satisfaction and, more importantly, correct identification of healthful foods [22, 23]. However, it is not yet known whether such an approach influences actual purchases, and it has yet to be empirically tested in restaurant or cafeteria settings. In the present intervention, every food item in the hospital cafeteria and vending machines was labeled with calories, number of steps required to burn those calories (e.g., a slice of pizza = 690 calories and 13,800 steps), and a “traffic light” color rating (green = go, eat in large portions, yellow = caution, eat in moderate portions, red = stop, eat in small portions; see Fig. 2). Color codes were based on energy density, which is the number of kcalories per gram of food (green ≤ 1.0 kcal/g; yellow = 1.0 to 2.25 kcal/g; red > 2.25 kcal/g. This scheme was based on Volumetrics principles [24], but adapted to fit a 3-color traffic light model, rather than the four categories initially proposed by Rolls and Barnett. Most fruits and vegetables are “green foods,” whereas high-fat foods are “red foods” because fat has more than twice the number of calories per gram than carbohydrates or proteins. Step equivalents were based on the general principle of 100 kcalories expended per mile of walking (an individual weighing 175lbs, walking 4 mph, would burn 99 calories [25]). It was anticipate that such a labeling scheme at the point-of-selection would provide a highly salient and meaningful visual cue as to the availability of healthful foods, as well as affect employees’ eating behavior by changing their knowledge of and self-efficacy toward eating lower calorie food while increasing the perceived risk of eating high calorie foods. To our knowledge, no study has ever examined the effects of this particular labeling scheme.

#### Environmental changes in cafeteria

Over the course of the study, a number of changes were made to the hospital cafeteria. Due to the difficulty of making these types of environmental changes at an institution, most of these modifications did not occur until the latter half of the study. Changes included reducing the size of serving spoons (start of study), offering half portions at half price (at month 5), increasing the number of “green light” foods (month 7), moving the dessert case to a less visible area (month 8), establishing a highly visible “grab-n-go” cooler offering healthy foods (month 8), and decreasing portion sizes of some foods (month 8). These changes were designed to increase perceptions of access to and convenience of healthy foods, and to make the default the healthier option.

#### Pedometers

It has been increasingly recognized that to promote and maintain physical activity habits, individuals need to identify and make use of activities that can be readily adapted to their environments and schedules [26]. Because walking is a convenient and inexpensive form of physical activity, it offers a promising strategy for altering sedentary behaviors and managing weight. Worksite walking programs utilizing pedometers have produced increases in moderate intensity physical activity levels as well as improvements in BMI, systolic blood pressure, blood glucose, and cholesterol [27–29].

As part of the Go! study, participants who completed the baseline assessment received a pedometer (Digi-Walker SW-200) that tracked number of steps per day, and were given a brochure containing information about its use, including its relation to the Go! program. Participants were informed about the labeling scheme in the cafeteria that was about to be initiated, and told how the pedometer could help them monitor activity in relationship to food intake (i.e., energy balance). In addition to providing an increase in awareness/knowledge of one’s physical activity level, access to pedometers were expected to (a) create more positive attitudes towards walking, such that employees would view walking as a convenient and inexpensive form of physical activity; and (b) increase self-efficacy toward maintaining a walking program by giving employees a tool to easily self-monitor and adjust their amount of walking, especially in response to calories consumed (as marked in the cafeteria). These changes in attitude and self-efficacy were, in turn, expected to motivate employees to increase their actual (walking) behavior. For instance, employees with a pedometer may be more motivated to take the stairs instead of the elevator at the worksite. Complementary informational and motivational messages were offered to help employees reduce barriers to walking at work (i.e., signage pointing out the stairs, mapping walking routes).

Participants were provided with a personalized magnet providing energy balance facts and the number of calories each individual needed per day to maintain their current weight. It was hoped that the magnet would be placed in a frequently viewed location (refrigerator door, work filing cabinet), thereby increasing familiarity with energy balance principles, serving as a prompt to consider healthy eating and activity behaviors, and facilitate branding of the Go! program.

#### Environmental strategies to increase physical activity

A stairwell campaign incorporated stair use signs adapted from the CDC’s StairWELL campaign [30] as well as signs created specifically for *Go!* that were placed in and around stairwells and elevators. Two indoor walking routes were created, along with posters and flyers of maps, distances, and step information. These materials developed for this intervention component were designed to target the key constructs of knowledge, self-efficacy, social norms, access, and convenience.

#### Persuasive messaging

Strategic, theoretically derived, health communication messages aimed at changing employee attitudes, risk perceptions, and efficacy were offered in a variety of mediums including posters, table toppers, and a website. Messages were placed in and around stairwells, the cafeteria, break rooms, and hallways. There were three phases in message design and dissemination. In the initial phase, messages focused on educating employees about the meaning of the "traffic light" labels affixed to foods in the cafeteria as well as the calorie content of popular foods. Phase 1 messages were designed to motivate employees to eat more green light foods and cut back on red light foods. The goal of the second phase was to educate employees about the meaning of energy balance and the importance of portion sizes. In this phase, messages were also designed to motivate employees to use the stairs, pedometer, gym, and walking routes and to encourage self-efficacy with tag lines such as “even small changes, make big differences.” The final phase focused on underscoring the role of social support in losing and maintaining weight-loss both as a provider and recipient of social support. This phase was designed to coincide with the influential component of the intervention (described below). An additional file depicts examples of messages used in the *Go!* campaign (see Additional file 1).

#### Influentials

Another novel component of the Go! study was the use of “influentials” or natural helpers to influence social norms and enhance social support within the worksite. Influentials are well-respected, knowledgeable, socially well-connected and persuasive employees [31, 32] who are able to affect others’ attitudes and behaviors [33], thus providing a sustainable change to social norms. Studies point to the importance of strong and supportive social ties in influencing health-related behaviors, especially in relation to weight control [34] and dietary change [35]. A number of worksite health promotion programs also note the importance of social support and the social culture in which eating takes place in enabling health behavior change [3, 36].

In this study, influentials were identified and trained to provide support, reinforce healthful messages, change perceptions of risk, influence positive attitudes, and increase self-efficacy of employees, thereby complementing the messages on receipts and other materials. Notably, influentials provide an advantage over mediated messages in that influentials are able to deliver more personalized and tailored messages to each person in their worksite social network. Through informal channels of communication, the influential can address and clarify misconceptions or gaps in information that particular people may have and encourage others to change behaviors.

Influentials for the Go! study were identified in two primary ways. The first involved an employee survey completed by 530 out of 1565 hospital employees (34 % response rate). The survey included a validated measure that included the Maven scale (measuring health knowledge), the Persuader scale (measuring persuasiveness), and the Connector scale (measuring connectedness to others in the hospital) [37]. Participants were also asked to list fellow employees who met the description of a connector, persuader, and/or maven. Secondly, supervisors and directors (*n* = 61) were asked to identify employees who exemplified these three key characteristics.

Of the 83 eligible employees identified as meeting the characteristic of an influential, 54 agreed to participate (65 % acceptance rate). An initial 4-h training emphasized listening and persuasion skill-building in addition to providing basic education of energy balance principles and team building. Three, 1-h follow-up sessions included training in motivational interviewing, identification of barriers to healthy eating and physical activity among employees, and development of strategies to reduce barriers. The influentials were encouraged to develop a plan to move forward, beyond the 12-month study timeframe. This resulted in the formation of action committees who chose to target environmental and policy changes (e.g., policies limiting free lunches from pharmaceutical representatives, initiating exercises classes on site). Such broader changes were seen after the conclusion of the study period. Further details of the selection procedures, trainings, and assessment related to this component were published elsewhere [38].

### Fidelity checks

Intervention fidelity was assessed in a number of ways. All cafeteria food labels were checked by study staff for accuracy at the start of each weekday lunch period (the busiest period of use among employee patrons) and continued through the entire 12 months. Vending machine labels were verified weekly and any new items were added to the labels within one week. Weekly to biweekly fidelity checks were conducted to assure that mediated messages were posted (posters hung and visible in stairwells, elevators hallways, and break rooms; table tents were on each cafeteria table and in break rooms).

### Measures

Employees completed assessments at baseline, 6-months, and 12-months. Questionnaires were sent out (hard copy and/or electronically with link to online version) in advance of data collection and took approximately 30 min to complete. Participants then underwent anthropometric measurements completed by trained research personnel in a private clinic room or an enclosed area within the cafeteria. Several data collection sessions were scheduled over a 3-week period, offering a variety of times to accommodate hospital work shifts. Many of the measures were selected to be consistent with those in the NHLBI-funded studies [6], so comparisons could be made.

#### Anthropometric measures

Weight was measured in light clothing and without shoes to the nearest 0.2 lbs using a standard scale equipped with a leveling bubble on the platform (Seca 869). Height was measured without shoes to the nearest 0.25 in. using a calibrated portable stadiometer (Charder HM200p Portstad). BMI was calculated as weight (kg) / height^2^ (m). Waist circumference was measured to the nearest 0.1 cm using a standard tape equipped with a tension calibration mechanism (Gulick II). Measurement procedures were adapted from the National Health and Nutrition Examination Survey III - Anthropometry Procedures Manual [39]. Two waist measurements were taken, and the average used. If the difference was greater than 0.5 cm, a third measurement was taken, and the average of the two closest scores was used.

#### Dietary intake

The National Cancer Institute Multifactor Screener [40] is a 17-item self-report food frequency questionnaire that estimates daily fruit and vegetable intake, grams of fiber, and percent of energy from fat. Out of 9 frequency options ("*never*" to "*4 or more times per day*"), employees selected the one that best estimated specific food intake over the past month. Scores on all three variables have been found to be highly correlated (*r* = 0.5–0.8) with estimates of actual intake [41]. This screening measure was selected because it was expected to be most sensitive to changes in eating behavior targeted by the intervention (i.e., fruits and vegetables are low-calorie, low energy-dense foods and thus encouraged, whereas high fats foods were discouraged due to their higher calories and energy density). Other foods specifically targeted in *Go!* (including donuts/muffins, cookies/bars, sugary beverages, and ice cream, all served in the cafeteria) were added to the survey. These items were analyzed separately and not included in the subscale calculations.

#### Physical activity

Two self-report measures were used to gather data about physical activity. Found to be both reliable and valid [42], the Godin Leisure Time Exercise Questionnaire (GLTEQ) [43] is a brief measure that asks an individual about weekly frequencies of strenuous, moderate, and light activities, and yields an overall intensity-weighted frequency score [metabolic equivalent per week (METs)]. The International Physical Activity Questionnaire (IPAQ) [44] walking subscale was also used. Based on the work of French and colleagues [45], modifications were made to shorten the instructions and simplify response formats. Given that increasing walking as part of one’s daily routine was a specific target in the Go! intervention, the measure was further shortened to four items that only assess minutes per day of walking across work, home, and leisure settings (with a total score calculated). Participants were asked to select one of eight frequencies (e.g., less than 10 min, 10–20 min…more than 2 h).

#### Knowledge

A 6-item measure designed to assess knowledge about principles of energy balance was created for this study. Items included individualized information (e.g., *“How many calories do you think YOU need each day to maintain your current weight?”*), general information (e.g., number of calories needed to cut in order to lose one pound), and calorie content in foods commonly purchased in the hospital cafeteria (e.g., regular fat salad dressing).

#### Perceptions of support

From the Worksite Health Climate Scale [46], the 4-item Organizational Support Scales of Employer’s Health Orientation assessed how employees perceived employer concern about the health and well-being of employees (e.g., *“[Hospital Name] values workers who have healthy lifestyles.”*, and the 3-item Job Flexibility to Exercise subscale assessed perceptions that one’s job is flexible enough to allow exercise at work (e.g., “*Í can make time to exercise at some point during normal work hours.”)*. The items were rated on a 5-point response format (1 = *strongly disagree* to 5 = *strongly agree*) with a mean item value calculated for each of the subscales. Internal consistency was adequate in the present study (Commitment, *α* = 0.87–0.89; Flexibility, *α* = 0.70–0.77).

#### Perceived coworker support

This 3-item measure, adapted from the Route H study [45], asked employees about support they received from coworkers with respect to eating a low-calorie/low-fat diet, exercise, and weight management (e.g., *“How supportive are your coworkers of your efforts to manage your weight?”)*. Scores ranged on a 6-point scale from “*not applicable,*” “*not at all supportive*” to “*very supportive.*” The three items were added for a total score. The measure demonstrated good internal consistency in this study (*α* = 0.93).

#### Information

Three items assessed access to information about healthy eating, physical activity, and weight management on a 7-point scale (e.g., *“There is a lot of information on healthy eating at [hospital name]”*. The scale was adapted from the Route H study [45] and had high internal consistency (*α =* 0.92–0.96).

#### Reactions to *Go!*

On the final survey, participants were asked questions about their attitude toward, use of, and desire for certain components of the intervention to continue.

#### Weight and health history

Twenty-three questions regarding weight goals, weight loss history, and health and medication status were also included.

### Statistical analysis

Data were analyzed using SAS software, version 9.4 and SPSS, version 21. Missing data for outcome variables and covariates were imputed using fully conditional (MCMC) multiple imputation. Imputations were run separately for sets of variables expected to strongly related to each other and separately for each campus. SAS procedure MIANALYZE was used for combining of analyses across multiply imputed data sets.

For the intervention group, the number of missing values that were multiply imputed increased across the three time points (1.7, 20.3 and 21.9 %, respectively); and were almost entirely from complete missing assessments at follow up time points. For the comparison group 1.2, 10.7 and 8.5 % of data values imputed at each of the time points, respectively. When residuals from outcome variables were highly skewed (i.e., weight, BMI, waist circumference, IPAQ, GLTEQ, dietary screen variables), the natural log was used for analyses. Results are reported in the original scale means or originals scale geometric means for ease of interpretation.

Comparisons of baseline differences were made between participants in the intervention versus comparison group using *t*-tests for continuous variables and either Chi Square or Fisher’s exact test for categorical variables. These same tests were used to compare participants who completed two or three anthropometric assessments (completers) versus those who completed only baseline assessments (noncompleters).

The impacts of the intervention on the outcome variables were assessed using repeated measures ANCOVAs (adjusted for known correlates of BMI and other reported measures, including age, sex, education, income, smoking status, job category, perceived job flexibility, weight loss goal, and being on medications for diabetes, depression, hyperlipidemia, or hypertension). For covariates that could change over time, the covariate value at each time point was used as the covariate for the dependent variable at that time point.

For all repeated measures analyses, data were also analyzed using nonimputed, reported data only, and by using the last observation carried forward (LOCF) method for handling missing data as an additional intent-to-treat approach. The results of all six analyses (nonimputed, multiply imputed, and LOCF; each with and without covariates) were similar with a few differences for which comparisons were slightly above or below *p* = 0.05. The analyses reported here are from the multiply imputed data with covariate adjustments. For all outcomes, changes in adjusted and unadjusted means were compared over time within groups, and changes from baseline were compared across groups with interaction type contrasts (i.e. Time 2 - baseline for intervention group versus Time 2 - baseline for comparison group, and similarly for Time 3 - baseline.

## Results

### Enrollment and retention

**Fig. 3:**
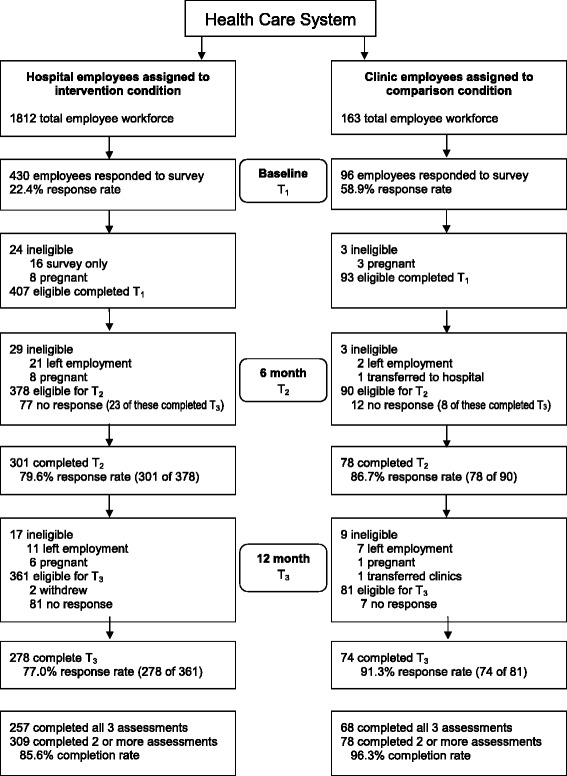
Individual participant recruitment flow diagram

A total of 407 hospital employees in the intervention arm and 93 clinic eligible employees in the comparison arm were assessed at baseline (22.4 % and 58.9 % response rate, respectively). Of participants remaining eligible, 309 out of 361 hospital employees (85.6 %) and 78 out of 81 clinic employees (96.3 %) completed the 6- and/or 12-month assessment (i.e., “completers”). Figure 3 shows study enrollment and retention. Pregnant women (*n* = 25) were excluded from all analyses as were individuals who left employment (*n* = 43) or moved between hospital and clinic settings (*n* = 3), and were not considered to be “noncompleters.”

### Participants

The majority of participants were white (92.5 %), female (85.1 %), and married (66.5 %). Mean age was 43.0 ± 11.7 years with a mean BMI of 28.4 ± 6.3 kg/m^2^ (33.0 % overweight, 33.3 % obese). Employees providing direct patient care represented about half (51.3 %) of the sample. See Table 1 for a description of the samples. In general, the sample represented the healthcare system’s population (e.g., 79 % female, 96 % white), though service and administration employees were overrepresented, and patient care providers, particularly physicians, were underrepresented.

**Table 1:** Baseline comparison of intervention vs. comparison group and completers vs. noncompleters

Variables	Intervention (*n* = 361)^b^ *n* (%) or Mean (SD)	Comparison (*n* = 81)^b^ *n (*%) or Mean (SD)	*p* ^a^	Completers (*n* = 387)^c^ *n* (%) or Mean (SD)	Noncompleters (*n* = 55)^c^ *n* (%) or Mean (SD)	*p* ^a^
Demographic Variables						
Age (y)	43.0 (12.1)	43.3 (9.8)	0.82	43.3 (11.6)	41.3 (12.8)	0.25
Gender (female)	298 (82.6 %)	78 (96.3 %)	0.002	329 (85.0 %)	47 (85.5 %)	0.93
Race/ethnicity (White)	329 (91.9 %)	77 (95.1 %)	0.08	352 (91.7 %)	54 (98.2 %)	0.47
Education			0.18			0.61
High school or less	46 (12.9 %)	12 (15.0 %)		53 (13.9 %)	5 (9.3 %)	
Some college or vocational training	127 (35.7 %)	36 (45.0 %)		143 (37.4 %)	20 (37.0 %)	
College degree +	183 (51.4 %)	32 (40.0 %)		186 (48.7 %)	29 (53.7 %)	
Annual household income			0.55			0.21
< 30,000	58 (17.1 %)	13 (17.3 %)		59 (16.2 %)	12 (24.0 %)	
30,000–59,999	101 (29.8 %)	24 (32.0 %)		112 (30.8 %)	13 (26.0 %)	
60,000–100,000	116 (34.2 %)	29 (38.7 %)		132 (36.3 %)	13 (26.0 %)	
> 100,000	64 (18.9 %)	9 (12.0 %)		61 (16.8 %)	12 (24.0 %)	
Job category			< 0.001			0.45
Patient care	182 (51.3 %)	40 (51.3 %)		191 (50.3 %)	31 (58.5 %)	
Administrative/clerical	103 (29.0 %)	38 (48.7 %)		125 (32.9 %)	16 (30.2 %)	
Service	70 (19.7 %)	0 (0 %)		64 (16.8 %)	6 (11.3 %)	
Smoker (yes)	37 (10.5 %)	10 (12.7 %)	0.57	38 (10.0 %)	9 (17.0 %)	0.13
Primary outcome variables						
Anthropometrics						
Weight (kg)	77.6 (18.1)	76.5 (18.9)	0.62	76.9 (17.8)	81.0 (20.9)	0.12
BMI	28.4 (6.2)	28.4 (6.6)	0.93	28.2 (6.2)	29.6 (7.2)	0.12
Waist circumference (cm)	92.9 (14.9)	94.7 (17.6)	0.36	92.8 (15.2)	96.2 (16.8)	0.13
Dietary measures (per day)						
Fruit/vegetable servings	5.2 (2.7)	4.5 (2.2)	0.04	5.1 (2.6)	4.8 (2.7)	0.50
% calories from fat	32.3 (6.0)	31.0 (5.7)	0.08	32.2 (6.1)	31.4 (5.3)	0.34
Fiber (g)	20.9 (14.6)	18.1 (10.6)	0.11	20.3 (13.7)	21.2 (15.9)	0.65
Physical activity measures						
GLTEQ	42.5 (31.8)	37.9 (28.9)	0.23	41.9 (30.6)	39.5 (35.9)	0.59
IPAQ	137.3 (89.5)	113.8 (68.7	0.03	132.3 (86.1)	138.2 (89.6)	0.64
Secondary outcome variables						
Knowledge	1.2 (1.1)	1.3 (1.2)	0.36	1.2 (1.1)	1.4 (1.3)	0.35
Employer commitment	3.5 (0.7)	3.6 (0.6)	0.56	3.5 (0.7)	3.5 (0.7)	0.87
Coworker social support	8.3 (4.7)	9.3 (4.0)	0.04	8.6 (4.6)	7.8 (4.7)	0.23
Information availability	4.1 (1.4)	3.0 (1.3)	< 0.001	3.8 (1.5)	3.9 (1.6)	0.64
Health discussions with coworkers	2.9 (1.4)	3.8 (1.5)	< 0.001	3.0 (1.5)	3.3 (1.3)	0.19

^a^ *t*-tests were used for continuous variables and chi-square or Fisher’s exact tests were used for categorical variables

^b^Data reported are from employees who remained eligible throughout the study

^c^“Noncompleters” were defined as those who did not complete any assessments after baseline; “completers” completed two or all three assessments

*GLTEQ* Godin Leisure Time Exercise Questionnaire, *IPAQ* International Physical Activity Questionnaire

At baseline, the intervention group had fewer females, fewer participants in administrative/clerical positions, and more participants in service positions than the comparison group. These differences are consistent with the overall makeup of each worksite. The groups had similar anthropometric measures at baseline, but differences in some of the other measures. The intervention group reported more fruits and vegetables consumed, greater amounts of weight management information available, higher levels of walking, less perceived coworker social support, and fewer health discussions with coworkers. Differences on these latter three variables are likely related to the work environment. The primary care clinics are smaller in size (so shorter distances to cover while walking at work) and number of staff (so more “close-knit”). Because the groups were nonequivalent at baseline, it seems particularly important to consider the within group changes over time in addition to the between group changes. Completers (*n* = 387) and noncompleters (*n* = 55) were similar on all baseline measures, so there was no apparent systematic reason for not remaining in the study.

### Intervention fidelity

The intervention was implemented as planned. Weekly to biweekly fidelity checks of mediated messages indicated that less than 1 % of messages were found to be missing overall. All new items in vending machine received labels within one week. Consistent posting of nutrition labels for all foods in the cafeteria posed the greatest difficulty; whereas foods offered daily had more permanent signage, many foods were offered only occasionally and thus labels needed to be updated daily. All hospital cafeteria staff were trained on how to find and post pre-made labels, and while some staff took ownership of this intervention component, others were less involved and did not contribute to assuring label accuracy. Because cafeteria cooks had the freedom to deviate from the posted menu and/or add new foods and recipes, there were occasions when a label was not available for posting. On average, over 95 % of foods were labeled on any given weekday.

In terms of the influential component of the intervention, all except one of the 54 influentials attended the initial two training sessions. Thirty-seven (69 %) attended at least one of the three follow-up meetings and 22 (41 %) attended two or all three. Three peer helpers requested to discontinue participation during the program. It is difficult to assess the degree of fidelity with which the influentials acted upon their prescribed role, as they were not assessed during the study on their acquisition of knowledge or skills on which they were trained. However, qualitative data from interviews with a subset of 20 influentials suggest that the role of a “peer helper” was embraced by most participants [38]. These influentials reported modeling recommended behaviors and having more conversations with their coworkers about healthy eating and physical activity; although they were more comfortable with some topics (e.g., “Let’s take the stairs.”) than with others (e.g., “Let’s not buy that ‘red’ food.”).

### Effects of the intervention on primary outcomes

The adjusted, imputed, descriptive data are presented in Table 2. Results from repeated measures ANCOVA appear in Table 3. There were generally no significant changes in weight or BMI between the groups or over time. Employees in the comparison group had a greater decrease in waist circumference at 6-months than the intervention group (*p* = 0.001), but not at 12-months.

**Table 2:** Adjusted means and standard errors for groups at baseline, 6 months, and 12 months

	Baseline	6 months	12-months
Variables	M (Se)	M (Se)	M (Se)
Weight (kgs)			
Intervention	75.51 (0.84)	75.21 (0.83)	75.71 (0.83)
Comparison	74.58 (1.97)	74.14 (1.97)	75.03 (2.08)
BMI^a^			
Intervention	27.77 (0.29)	27.74 (0.29)	27.87 (0.29)
Comparison	27.50 (0.66)	27.17 (0.68)	27.55 (0.71)
Waist (cm)^a^			
Intervention	91.65 (0.71)	91.61 (0.74)	91.51 (0.72)
Comparison	93.43 (1.80)	90.68 (1.66)	92.79 (1.79)
IPAQ^a^			
Intervention	108.98 (3.62)	118.01 (3.95)	122.50 (4.63)
Comparison	101.97 (7.12)	112.35 (8.09)	93.64 (6.68)
GLTEQ^a^			
Intervention	32.71 (1.62)	39.02 (1.95)	35.30 (1.64)
Comparison	28.96 (3.25)	36.59 (3.67)	28.87 (3.21)
Fruit & vegetable servings^a^			
Intervention	4.46 (0.13)	4.70 (0.13)	4.11 (0.13)
Comparison	4.07 (0.22)	4.59 (0.25)	4.25 (0.26)
% calories from fat^a^			
Intervention	31.82 (0.33)	31.50 (0.39)	31.62 (0.41)
Comparison	30.57 (0.69)	30.78 (0.66)	31.23 (0.73)
Fiber (g) daily^a^			
Intervention	17.70 (0.49)	18.76 (0.54)	16.54 (0.43)
Comparison	16.68 (0.89)	17.99 (0.99)	17.55 (1.16)
Knowledge			
Intervention	1.21 (0.06)	1.57 (0.07)	1.59 (0.08)
Comparison	1.32 (0.13)	1.42 (0.12)	1.48 (0.14)
Information			
Intervention	4.04 (0.07)	4.77 (0.08)	4.96 (0.09)
Comparison	2.96 (0.15)	3.29 (0.16)	3.27 (0.15)
Employer commitment			
Intervention	3.49 (0.04)	3.60 (0.04)	3.63 (0.04)
Comparison	3.57 (0.07)	3.57 (0.08)	3.52 (0.08)
Coworker social support			
Intervention	8.84 (0.21)	8.82 (0.24)	8.61 (0.26)
Comparison	9.26 (0.40)	9.15 (0.36)	8.87 (0.41)
Health discussions with coworkers			
Intervention	2.91 (0.07)	3.24 (0.14)	3.08 (0.09)
Comparison	3.57 (0.16)	3.36 (0.17)	3.18 (0.17)

*GLTEQ* Godin Leisure Time Exercise Questionnaire, *IPAQ* International Physical Activity Questionnaire

^a^Geometric means and standard error calculated from natural log transformed values

**Table 3:** Changes in outcome variables at 6 and 12 months

	Change baseline to 6 months within groups		Group Differences		Change baseline to 12 months within groups		Group Differences	
Variables	Change (% or score^a^; 95 % CI)	*p*	Mean difference (% or score^a^; 95 % CI)	*p*	Change (% or score^a^; 95 % CI)	*p*	Mean difference (% or score^a^; 95 % CI)	*p*
Weight (kgs)^a^								
Intervention	−0.39 % (−0.96, 0.18)	0.17	0.21 % (−1.13, 1.56)	0.76	0.27 % (−0.36, 0.89)	0.40	−0.32 % (−1.80, 1.17)	0.66
Comparison	−0.59 % (−1.69, 0.51)	0.29			0.59 % (−0.65, 1.85)	0.35		
BMI^a^								
Intervention	−0.11 % (−0.63, 0.40)	0.67	1.10 % (−0.17, 2.39)	0.09	0.34 % (−0.27, 0.95)	0.27	0.16 % (−1.18, 1.52)	0.81
Comparison	−1.20 % (−2.31, −0.07)	0.04			0.18 % (−1.07, 1.44)	0.78		
Waist (cm)^a^								
Intervention	−0.04 % (−0.64, 0.57)	0.90	2.99 % (1.28 to 4.72)	0.001	−0.15 % (−0.85, 0.56)	0.68	0.54 % (−1.06, 2.16)	0.51
Comparison	−2.94 % (−4.42, −1.44)	0.000			−0.68 % (−2.08, 0.73)	0.34		
IPAQ^a^								
Intervention	8.29 % (0.71, 16.44)	0.03	−1.71 % (−15.69, 14.59)	0.83	12.41 % (4.09, 21.38)	0.004	22.41 % (6.02, 41.32)	0.006
Comparison	10.17 % (−3.58, 25.89)	0.15			−8.17 % (−18.72, 3.74)	0.17		
GLTEQ^a^								
Intervention	19.30 % (6.80, 33.25)	0.003	−5.57 % (−25.09, 19.03)	0.63	7.93 % (−2.52, 19.50)	0.14	8.28 % (−13.73, 35.91)	0.49
Comparison	26.34 % (2.04, 56.41)	0.03			−0.33 % (−18.75, 22.27)	0.98		
Fruit & vegetable servings^a^								
Intervention	5.31 % (−0.38, 11.33)	0.07	−6.46 % (−16.35, 4.60)	0.24	−7.84 % (−13.13, −2.23)	0.007	−11.70 % (−21.76, −0.35)	0.04
Comparison	12.59 % (2.36, 23.84)	0.02			4.38 % (−6.43, 16.43)	0.44		
% calories from fat^a^								
Intervention	−1.01 % (−3.38, 1.41)	0.40	−1.71 % (−6.28, 3.08)	0.47	−0.65 % (−3.47, 2.26)	0.64	−0.28 % (−.762, 2.38)	0.28
Comparison	0.71 % (−3.12, 4.69)	0.72			2.16 % (−2.47, 7.01)	0.36		
Fiber (g) daily^a^								
Intervention	5.97 % (0.05, 12.23)	0.05	−1.71 % (−11.02, 8.57)	0.73	−6.55 % (−11.29, −1.55)	0.01	−11.15 % (−21.29, 0.31)	0.06
Comparison	7.81 % (−0.70, 17.05)	0.07			5.18 % (−5.44, 16.99)	0.35		
Cookies, Cakes, Brownies								
Intervention	−0.08 (−0.14, −0.03)	0.003	0.06 (−0.04, 0.16)	.22	−0.07 (−0.12, −0.01)	0.02	−0.06 (−0.18, 0.07)	.38
Comparison	−0.14 (−0.23, −0.06)	0.0007			−0.01 (−0.12, 0.01)	0.86		
Muffins, donuts								
Intervention	−0.05 (−0.09, −0.02)	0.006	−0.07 (−0.112, 0.02)	.009	−0.08 (−0.12, −0.05)	<0.0001	−0.08 (−0.12, −0.03)	.002
Comparison	0.01 (−0.02, 0.05)	0.45			−0.01 (−0.04, 0.02)	.59		
Ice cream								
Intervention	0.01 (−0.04, 0.01)	0.32	−0.02 (−0.06, 0.03)	0.45	−0.02 (−0.04, 0.00)	0.04	−0.02 (−0.07, 0.03)	0.41
Comparison	0.01 (−0.03, 0.05)	0.78			0.00 (−0.05, 0.04)	0.89		
Sugar sweetened beverages								
Intervention	0.01 (−0.03, 0.01)	.54	0.02 (−0.02, 0.05)	0.29	−0.02 (−0.04, 0.00)	0.02	−0.01 (−0.04, 0.01)	0.38
Comparison	−0.02 (−0.05, 0.00)	.08			−0.01 (−0.03, 0.01)	0.32		
Knowledge								
Intervention	0.36 (0.22, 0.50)	0.000	0.27 (−0.01, 0.55)	0.06	0.38 (0.22, 0.54)	0.000	0.23 (−0.09, 0.54)	0.16
Comparison	0.09 (−0.15, 0.34)	0.45			0.15 (−0.12, 0.43)	0.27		
Information								
Intervention	0.73 (0.57, 0.90)	0.000	0.40 (0.08, 0.72)	0.02	0.92 (0.70, 1.14)	0.000	0.61 (0.24, 0.99)	0.002
Comparison	0.33 (0.05, 0.62)	0.02			0.31 (0.02, 0.60)	0.04		
Employer commitment								
Intervention	0.11 (0.03, 0.19)	0.006	0.10 (−0.05, 0.26)	0.19	0.14 (0.06, 0.23)	0.002	0.19 (0.03, 0.35)	0.02
Comparison	0.01 (−0.14,0.15)	0.92			−0.05 (−0.17, 0.08)	0.46		
Coworker social support								
Intervention	−0.03 (−0.53, 0.47)	0.92	0.09 (−0.87, 1.04)	0.86	−0.23 (−0.75, 0.29)	0.37	0.16 (−0.88, 1.21)	0.75
Comparison	−0.11 (−0.97, 0.75)	0.80			−0.39 (−1.30, 0.51)	0.39		
Health discussions with coworkers								
Intervention	0.33 (−0.02, 0.68)	0.06	0.54 (0.07, 1.01)	0.03	0.17 (0.00, 0.34)	0.05	0.56 (0.15, 0.97)	0.008
Comparison	−0.21 (−0.58, 0.17)	0.28			−0.39 (−0.78, −0.01)	0.05		

Note: Difference scores are based on means adjusted for age, sex, education, income, smoking status, job category, job flexibility, weight loss goal, and taking medication for depression, diabetes, hypertension, or hyperlipidemia

^a^Variables in which *p* values are based upon analyses using natural log transformations

There were no group differences in overall exercise (GLTEQ), but the intervention group reported greater increases in walking activity (IPAQ score) at 12-months than the comparison group (*p* < .006). Self-reported fruits and vegetables servings also did not significantly differ between groups over time, with the intervention group showing a statistically significant decline over 12 months (−0.35 servings/day, *p* = 0.007). Self-reported fiber intake had similar results, with a decline over 12-months for the intervention group. On the other hand, all four categories of high fat/sugar foods added to the diet measure (because they were a particular target of the intervention) revealed significant decreases (*p =* 0.02 to < 0.0001) at 12-months for the intervention group (not tabled).

### Effect of intervention on secondary outcomes

The intervention had significant effects for several secondary outcome measures. Employees in the intervention group showed improved knowledge of energy balance, increased perceptions for amount of employer information about healthy eating, physical activity and weight loss, increased perceptions of employer commitment and support to employees’ health and well-being, and increased health discussions with coworkers. These improvements in employer commitment, information, and health discussions were statistically (*p*’s = 0.02–0.008) greater than experienced within the comparison group. However, there were no significant changes in perceptions of coworker social support over time within or across groups.

### Perceptions of *Go!*

Employees in the intervention arm appeared highly satisfied with the *Go!* intervention. They expressed positive attitudes about the intervention and wanted all components of the intervention to continue, particularly those offered in the cafeteria (See Table 4).

**Table 4:** Intervention arm employees perceptions of *Go!*

Question	% hospital employees indicating “yes”
Would you like to see…	
the *Go!* program continue	86.8
continued display of nutrition labels	93.8
continued offering of ½ portions at ½ price	95.6
provision of pedometers	76.5
increased number of “green” foods	89.3
decreased number of “red’ foods	60.4
continuation of wellness messages	83.6
continuation/development of *Go!* website	73.3
Positive attitudes towards…	
nutrition labels	82.6
messages	83.8
Impact of *Go!* on behavior	
Not aware of the *Go!* program	0.7
No impact	26.6
Have made some changes	68.2
Have made substantial changes	5.2

## Discussion

This study evaluated a multi-component obesity prevention program (*Go!*) that used a social ecological framework within a workplace setting over a 12-month period. It is one of the first studies to implement nutritional labeling of all foods in a worksite cafeteria, and the first to use this type of salient labeling scheme. The primary hypotheses that the intervention would increase physical activity and healthier eating with accompanying prevention of weight gain were partially confirmed. There was no statistically significant difference in weight, BMI or waist circumference between groups over 12 months, though both the intervention and comparison groups gained less weight (.20 kg and .45 kg, respectively) than is expected among the adult population over the course of a year (i.e., ~0.82 kg) [47].

As expected, the intervention group showed an increase in daily walking time relative to the comparison group (approximately 22 min more per day). This finding is reinforced by supplemental data from participants in which the reported use of pedometers throughout the study increased from 4 % at baseline to 32 % at 12 months for the intervention group, whereas little change was noted in the comparison group over this same period (i.e., 3 % to 7 %). Moderate and vigorous levels of physical activity, however, remained largely unchanged. These findings are not entirely unexpected given the messages (e.g., “even small changes make a big difference,” calorie – step equivalency messages) and strategies that were used for increasing physical activity at the worksite (e.g., stairwell use, walking routes, pedometer distribution).

The reduction in fruit/vegetable and fiber intake and unchanged fat intake in the intervention group was disappointing, given that the nutritional labeling component of the intervention was focused on energy density. “Green light” foods with low energy density were primarily vegetables and fruits with high fiber content; thus we anticipated increases in both of these values. Subsequent analyses revealed, however, that total servings across all food categories in the screener went down in the intervention group (*t* = −4.04, *p* < 0.0001), but no such decrease occurred in the comparison group (*t* = 0.02, *p =* 0.98). It may be that the nutrition labels led hospital employees to cut calories regardless of “color.” Other potential explanations include possible insufficient sensitivity of the dietary assessment, lack of many “green” options in the cafeteria (and practically no such options in the vending machines), or recommended daily intake of 5 servings/day was already met at baseline and 6-months. Alternatively, the report of a high level of fruit and vegetable intake at baseline might be an overestimation, and that once employees were prompted to consider their intake, they may have become more aware of actual intake, and provided more accurate (lower) estimates of fruit and vegetable consumption as the study progressed. In future studies, a familiarization period might increase accuracy of reporting dietary intake. Finally, given fact that the high-fat/sugar foods added to our questionnaire (i.e., those specifically targeted in the intervention) decreased substantially, it may be that targeting specific foods may hold more promise than targeting food groups.

Changes over time and between groups with the secondary outcome measures were more evident and favorable. Knowledge about energy balance (e.g., “How many calories do you think YOU burn walking 1 mile?”) improved for the intervention group, as did perceptions about the amount of employer information pertaining to healthy eating, physical activity, and weight increased throughout assessments. Interestingly, the comparison group also perceived, albeit smaller in magnitude, an increase in this information. It may be that the comparison group felt they were getting more information by completing questionnaires and anthropometric assessments. Several participants from the clinics (comparison group) commented that they were excited to be part of a weight loss program, even though no intervention information was explicitly given to them from research staff. It may also be the case that they were aware of some changes occurring at the hospital as we only found perceptions of employer commitment to the health of employees increase with the intervention group. Employees in the intervention arm expressed positive attitudes about *Go!* and wanted continuation of changes, particularly those in the cafeteria.

A novel component of the intervention was to sway social norms through influential peers. Interviews with several influentials suggested that they had taken on many helping roles and saw themselves as role models to create health-promoting social and environmental changes at work [36]. These influentials also reported making behavioral changes themselves as they wanted to be role models and act in ways consistent with what they were encouraging from others [36]. Data from the current study indicated that indeed, the employees in the intervention group reported talking more with their coworkers about health related topics and that this change was seen across time as well as in comparison to the other group of employees. Despite this, there was no change in perceptions for the amount of coworker support they felt with respect to eating a low-calorie/low-fat diet, exercise, and weight management. Notably, we observed considerably higher levels of perceived coworker support for both groups (62 %–79 %) than those reported in a worksite study examining support among metropolitan transit employees (10 %–18 %) [41], suggesting that hospital employees may be amenable to interventions targeting existing social networks.

The current study has several strengths and limitations. As noted in a recent study [12], hospital employees are important targets for obesity prevention interventions as they may serve as models of health to the broader community, including patients. Additional strengths of *Go!* were the inclusion of multiple intervention components concurrently targeting dietary and physical activity, as well as novel elements with the use of traffic light labeling, and influentials. Also, all employees were allowed to participate in hopes of further influencing normative targeted health behaviors, versus selecting overweight employees or those wishing to lose weight. This broader inclusion, however, likely made detecting some changes in our primary outcome measures more challenging.

In terms of study limitations, relying on self-report may be subject to social desirability bias. Although we attempted to mitigate potential measurement error by incorporating widely used standardized measures, this is perhaps most problematic for assessment of physical activity and dietary intake, and may provide over/under-estimates for some individuals, but are expected to be the same for the intervention and comparison groups. Additionally, the study is limited by the lack of equivalency between hospital and clinic worksite settings and employees. There were baseline differences (gender, job type, fruit and vegetable consumption, daily walking, and coworker social support) between intervention and comparison groups. Although we controlled for this statistically, these differences limit our ability to draw definitive conclusions on effects of the intervention. Sample size also differed between groups; the smaller comparison group resulted in less power to detect significant group differences as well changes within the comparison group, than if the comparison group had been as large as the intervention group. We also observed some differences in response rates (higher for comparison group) and attrition rates (lower for comparison group). There seem to be understandable explanations for the greater participation with the comparison (clinic) group. The medical clinics were substantially smaller (*n* = 12–40), employees all worked day shifts, and one clinic manager facilitated the assessments. The intervention group (hospital) had multiple buildings, employees worked a variety of shifts, many employees had difficulty leaving patient care responsibilities, and there was no central, personal communication mechanism. Despite these challenges for hospital employees, it is important to note that the retention rate among the intervention group for those who remained employed and eligible was 86 % - an acceptable rate - and that differences are largely attributed to the exceptionally high rate among the comparison group (96 %).

Another limitation is that many of the environmental changes did not occur until the latter part of the intervention period. Changes that were easy to make (smaller serving spoons) happened quickly, as did any interventions directly entirely by research staff. On the other hand, those interventions requiring employer resources (installing a new electrical outlet in order to move a healthy food cooler to a more visible location) tended to be delayed, probably due to cost and levels of administrative approval needed. Similarly, changes that were feared to upset the employee customer, such as reducing portion sizes of desserts and creating a low-fat version of macaroni and cheese, were introduced slowly in order to assess degree of dissatisfaction that might occur.

## Conclusions

The effectiveness of a multi-method, broad reaching, but low-intensity social ecological intervention for weight gain prevention was not supported in this study in terms of amount of weight gain prevented, at least relative to the comparison group. Although most employees reported liking the intervention, making changes as a result of the intervention, walking more, and engaging in more discussions with coworkers about healthy eating and physical activity, this did not ultimately translate to prevention of weight gain beyond that seen in the comparison group. More research is needed to explore the effect of time on various modes of this approach. It may be that some intervention components have an immediate impact on behavior but decline when the novelty wears off, whereas other components may have more sustainable impacts. It may also be that long-term behavior change requires long-term exposure to individual, social, and environmental intervention strategies. For example, upon conclusion of the study, the influentials continued to push for policy changes (more bike racks, preventing pharmaceutical companies from providing food to employees) and the HR department eventually launched an employee walking competition that recruited a large percentage of the employees (which was a dramatic shift from past efforts). This suggests that a change in social norms took place, but perhaps was not yet developed by the end of the Go! intervention.

Greater attention to workers’ social networks as potential influencers of health practices is warranted. Changing dietary behaviors is complex, though targeting individual foods may be an effective approach. Also, implementing low-impact physical activity (e.g., walking, stair use) may prove to be more amenable to change and readily incorporated into the worksite setting than adding more vigorous forms of physical activity.

Finally, researchers considering worksite obesity prevention programs should be aware that there are substantial challenges in working with large organizations. Making significant changes to food systems, policies, and environments requires that key stakeholders are involved in (or at least highly support of) the design and implementation of intervention strategies that require resources from the employer (i.e., employee time, costs of materials). Administrators and managers must also be willing to make changes that may not initially be popular among employees).

## Additional file

Additional file 1:
**Example messages from the Go! campaign.** (PDF 2598 kb)
