# Supplementary material for: Go!: results from a quasi-experimental obesity prevention trial with hospital employees
Source: BMC Public Health. 2016 Feb 19;16:171. doi: 10.1186/s12889-016-2828-0 (PMC4759772; doi:10.1186/s12889-016-2828-0)
Supplement: Additional file 1: — Example messages from the Go! campaign. (PDF 2598 kb) [file 12889_2016_2828_MOESM1_ESM.pdf]

Phase 1

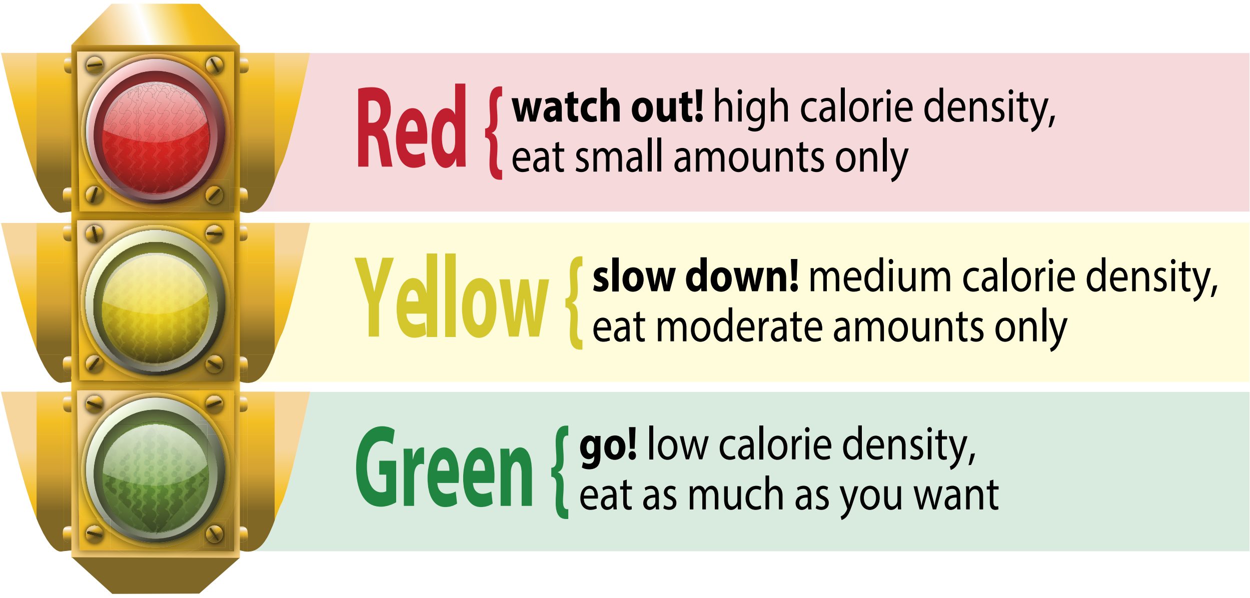

Red { watch out! high calorie density, eat small amounts only
Yellow { slow down! medium calorie density, eat moderate amounts only
Green { go! low calorie density, eat as much as you want

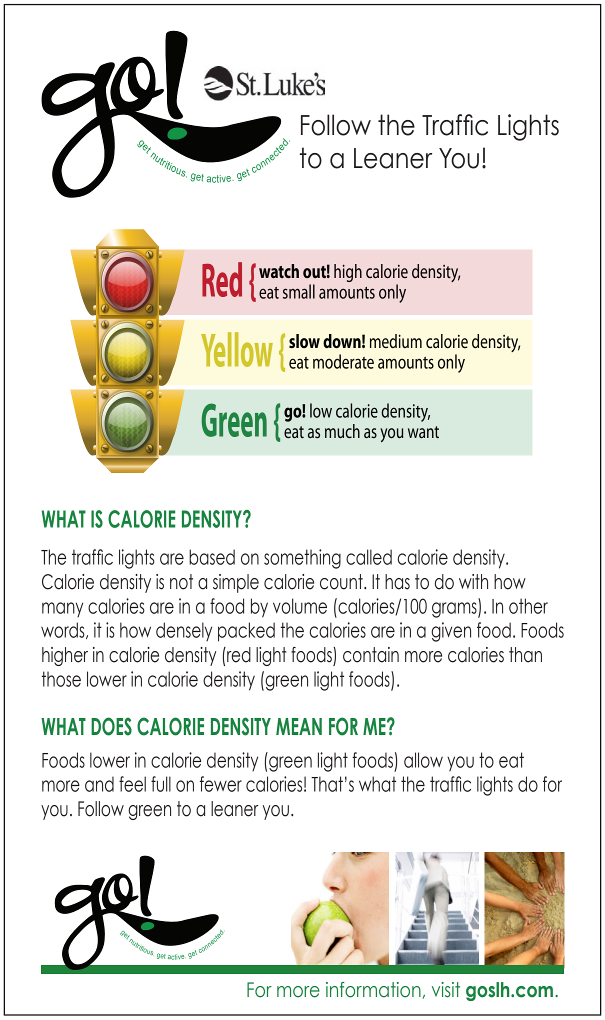

go!
get nutritious. get active. get connected.
St. Luke's
Follow the Traffic Lights to a Leaner You!
Red { watch out! high calorie density, eat small amounts only
Yellow { slow down! medium calorie density, eat moderate amounts only
Green { go! low calorie density, eat as much as you want
WHAT IS CALORIE DENSITY?
The traffic lights are based on something called calorie density. Calorie density is not a simple calorie count. It has to do with how many calories are in a food by volume (calories/100 grams). In other words, it is how densely packed the calories are in a given food. Foods higher in calorie density (red light foods) contain more calories than those lower in calorie density (green light foods).
WHAT DOES CALORIE DENSITY MEAN FOR ME?
Foods lower in calorie density (green light foods) allow you to eat more and feel full on fewer calories! That's what the traffic lights do for you. Follow green to a leaner you.
go!
get nutritious. get active. get connected.
For more information, visit goslh.com.

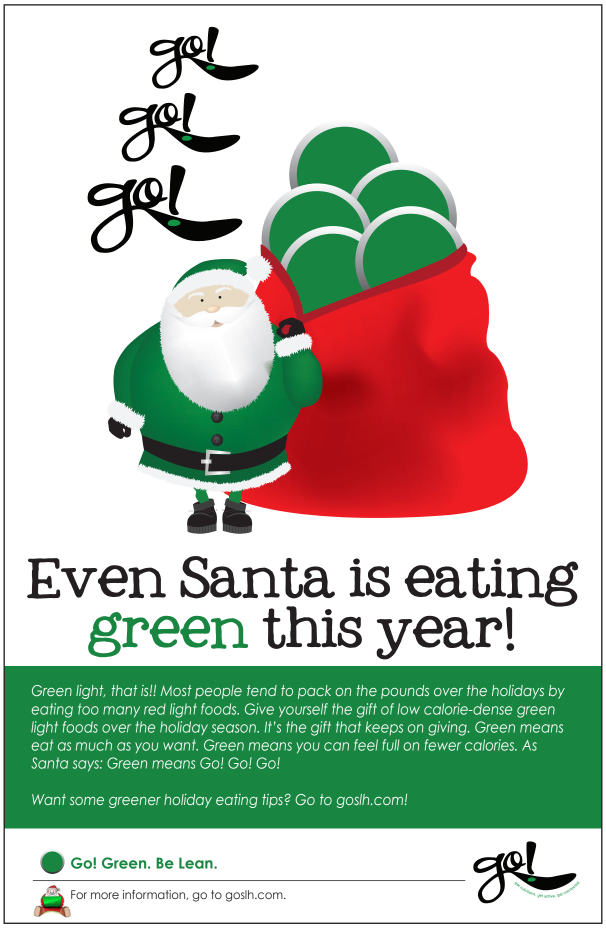

go!
go!
go!
Even Santa is eating green this year!
Green light, that is!! Most people tend to pack on the pounds over the holidays by eating too many red light foods. Give yourself the gift of low calorie-dense green light foods over the holiday season. It's the gift that keeps on giving. Green means eat as much as you want. Green means you can feel full on fewer calories. As Santa says: Green means Go! Go! Go!
Want some greener holiday eating tips? Go to goslh.com!
Go! Green. Be Lean.
For more information, go to goslh.com.
go!

Phase 2

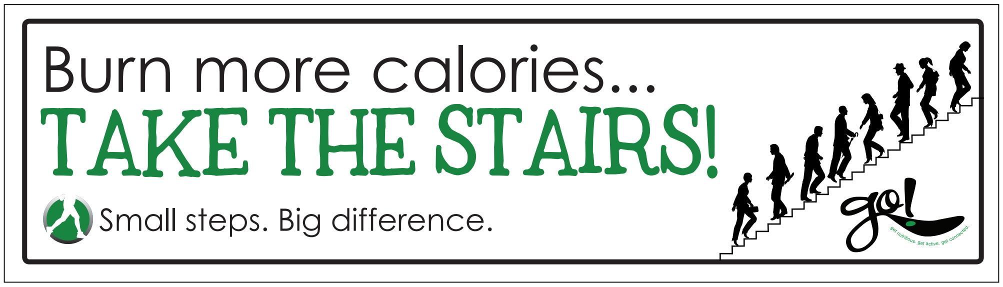
Burn more calories...
TAKE THE STAIRS!
Small steps. Big difference.

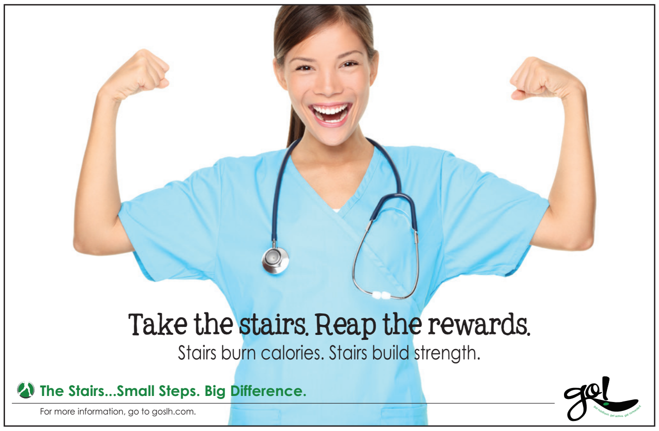
Take the stairs. Reap the rewards.
Stairs burn calories. Stairs build strength.
The Stairs...Small Steps. Big Difference.
For more information, go to goslh.com.

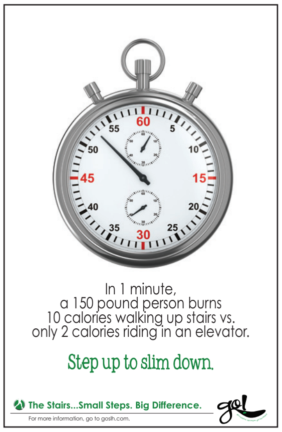
In 1 minute,
a 150 pound person burns
10 calories walking up stairs vs.
only 2 calories riding in an elevator.
Step up to slim down.
The Stairs...Small Steps. Big Difference.

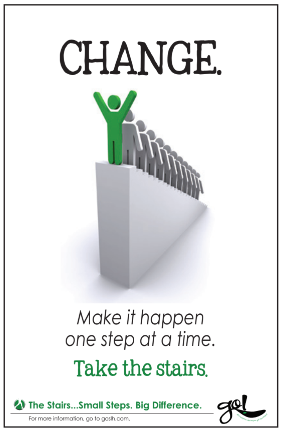
CHANGE.
Make it happen
one step at a time.
Take the stairs.
The Stairs...Small Steps. Big Difference.

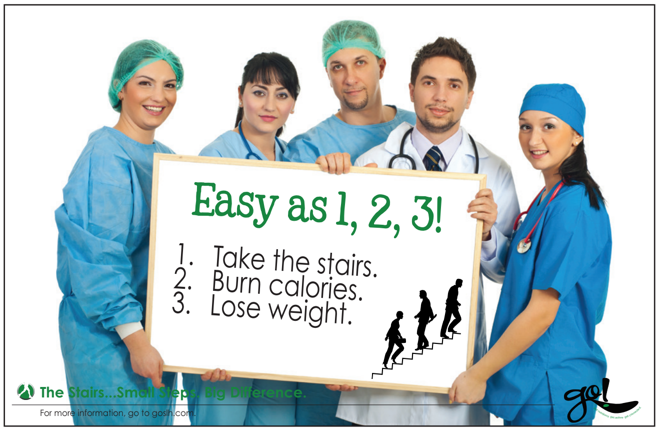
Easy as 1, 2, 3!
1. Take the stairs.
2. Burn calories.
3. Lose weight.

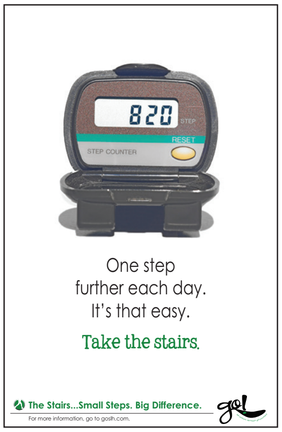
820
STEP COUNTER
RESET
One step
further each day.
It's that easy.
Take the stairs.
The Stairs...Small Steps. Big Difference.

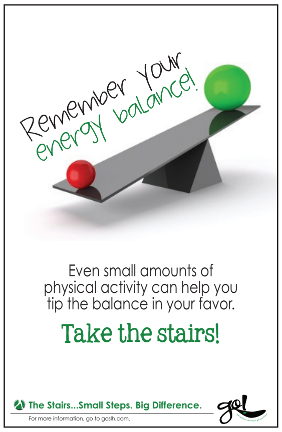
Remember your energy balance!
Even small amounts of
physical activity can help you
tip the balance in your favor.
Take the stairs!
The Stairs...Small Steps. Big Difference.

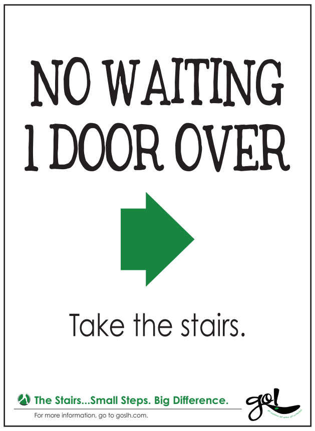
NO WAITING
1 DOOR OVER
Take the stairs.
The Stairs...Small Steps. Big Difference.

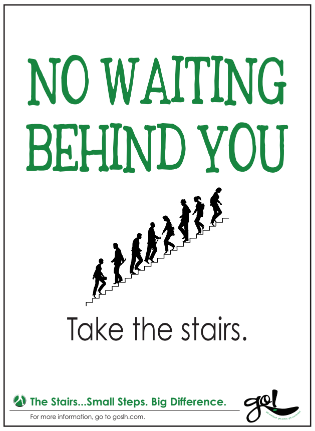
NO WAITING
BEHIND YOU
Take the stairs.
The Stairs...Small Steps. Big Difference.

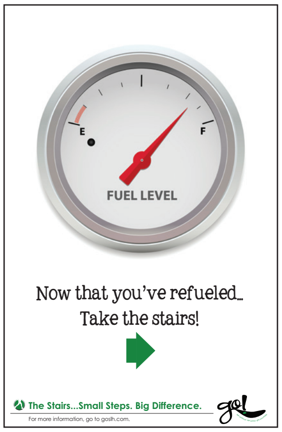
E
F
FUEL LEVEL
Now that you've refueled...
Take the stairs!
The Stairs...Small Steps. Big Difference.

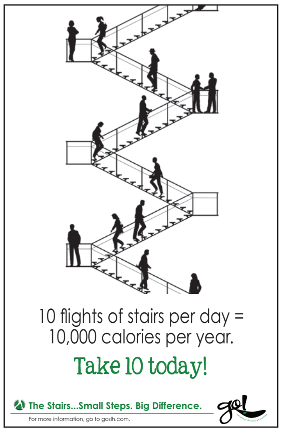
10 flights of stairs per day =
10,000 calories per year.
Take 10 today!
The Stairs...Small Steps. Big Difference.

Phase 3

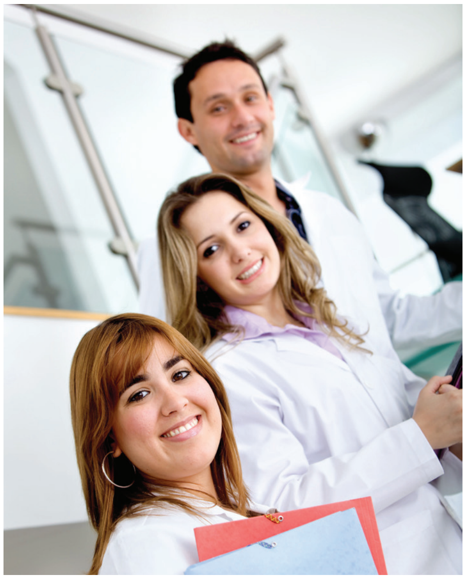

Build muscle & relationships.
Take the stairs with your friends.

The Stairs...Small Steps. Big Difference.

For more information, go to goslh.com.

go! get outdoors. get active. get connected.

You are not alone!

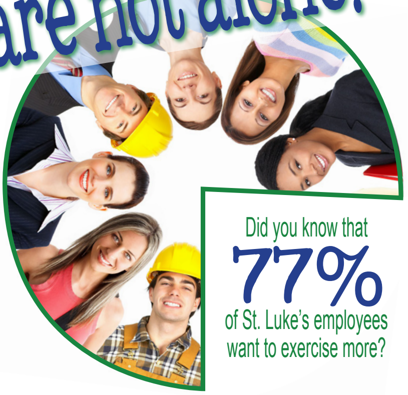

Did you know that
77%
of St. Luke's employees want to exercise more?

Want to exercise more than you do? Look around you. Three out of four of your co-workers want to do the same. The beauty is that you can help each other. Reach out and make exercise plans together. Your friends play a HUGE role in determining your weight loss (or gain)! Just get active together. Walk, run, hike, bike, camp, swim, ski, skate, play, compete, dance. Anything goes! Just Get Active!

Start now by grabbing a St. Luke's Walking Route Map and a friend...

Indoor walking routes tear-off pad here.<br>The Big Easy on the front.<br>The Little Easy on the back.	Outdoor walking routes tear-off pad here.<br>The Great Outdoors on the front.<br>The Mini Outdoors on the back.
INDOOR ROUTES	OUTDOOR ROUTES

Support Others. Support Yourself.

For information on fitness facilities, visit the GET FIT tab at goslh.com.

go! get outdoors. get active. get connected.

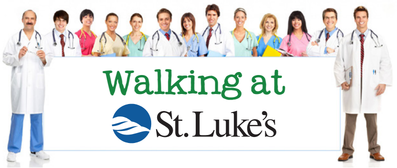

The Great Outdoors	Starting point: SLH Main Entrance	1 Lap= 1350 steps 70 calories	5 Laps= 3.5 miles 350 calories

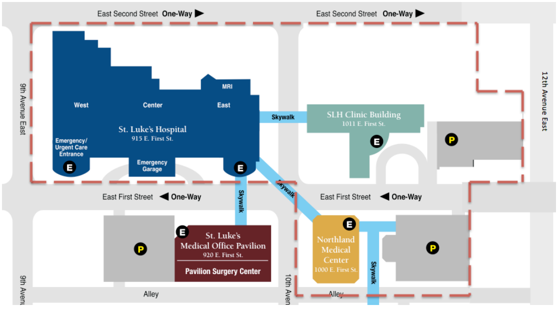

Small Steps. Big Difference.

For more information, go to goslh.com.

go! get outdoors. get active. get connected.
